# Gendered Social Norms, Exceptions, and Sanctions: Implications for Maternal, Infant, and Young Child Nutrition in Nigeria

**DOI:** 10.1016/j.cdnut.2024.104524

**Published:** 2024-12-10

**Authors:** Elizabeth Costenbader, Christina Memmott, Kate Litvin, Mackenzie Green, Nnenna Mba-Oduwusi, Izuchukwu Offiaeli, Nemat Hajeebhoy

**Affiliations:** 1 Global Health and Population Research, FHI 360, Durham, NC, United States; 2 Global Nutrition, Alive & Thrive, FHI 360, Washington, DC, United States; 3 InSiGHt Health Consulting Ltd, Abuja, Nigeria; 4 UNICEF Nigeria, Abuja, Nigeria

**Keywords:** nutrition, gender norms, meta-norms, maternal nutrition, infant and young child nutrition, male involvement, household decision-making, women’s mobility, community engagement, Nigeria

## Abstract

**Background:**

Nigerian pregnant and lactating women continue to experience high rates of malnutrition and Nigerian women experience long-term discrimination in the allocation and control of productive resources. Nigeria has policies and a governance architecture in place to advance nutrition, but these commitments lack recognition of how gender equity and nutrition are interwoven.

**Objective:**

To address this gap, this study sought to identify and analyze the influence of gender dynamics and gender norms on nutrition and health-related practices in Nigeria.

**Methods:**

This study used a combination of qualitative participatory activities and participant observation and questions to elicit information about the root causes of maternal, infant, and young child nutrition (MIYCN) and health concerns and typical nutrition and health practices in communities located across each of Nigeria’s 6 geopolitical zones. This analysis was informed by social norms theory and sought evidence of gender norms, as well as related social sanctions and norms exceptions.

**Results:**

Analysis of discussions with 503 participating men and women identified 3 overarching gender meta-norms that dictate women’s mobility, gendered delineation of household tasks, and gendered decision-making and, therefore, in turn influence women’s and men’s engagement in recommended MIYCN behaviors such as involvement in production and purchasing of nutritious foods and seeking healthcare and nutrition counseling. Participants also described strong traditional gender ideologies and sanctions that uphold these restrictive norms. Encouragingly, participants described some exceptions to norms, which could indicate changing gender norms.

**Conclusions:**

Nigeria currently has one of the highest rates of acute food and nutrition insecurity in Africa and contends with persistent gender inequalities. Interventions are needed that more explicitly tackle the links between gender inequality and nutrition. This analysis revealed gender meta-norms, and related sanctions and exceptions that affect multiple MIYCN behaviors and offer potential entry points for community engagement, programming, and policy around these issues.

## Introduction

Nutrition and gender are inextricably linked, and women and girls suffer disproportionately from poor nutrition. The 2023 UNICEF report “Undernourished and Overlooked” notes that women and adolescent girls are globally experiencing a “triple threat” of undernutrition, overnutrition, and micronutrient deficiencies [1]. Moreover, women are subject to social, cultural, and political restrictions on how food is produced, accessed and consumed, in addition to constraints on access and use of nutrition and health services [2].

This is true in Nigeria, where adolescent girls, pregnant and lactating women, and their young children continue to experience high rates of malnutrition [3]. Nearly half of all Nigerian women are either underweight or overweight (10% and 36%, respectively) and anemic (55%) and ∼1 in 2 women meet the minimum threshold for dietary diversity [3,4]. Nearly a third of children aged <5 y are stunted and 6.5% are wasted, with high rates of suboptimal feeding practices—34% exclusive breastfeeding, 31% minimum dietary diversity, 34% minimum meal frequency—reflecting gaps in information, support, and resources for mothers to adequately feed their children [3,5]. Nigerian women also experience long-term discrimination in the allocation and control of productive resources [6]. Nigeria has policies and a governance architecture in place to advance nutrition through a multisectoral approach, but these commitments lack recognition of how gender equity and nutrition are interwoven [7,8]. Without addressing this connection, it may prove difficult for Nigeria to meet the Sustainable Development Goal target of ending all forms of malnutrition by 2030 [9,10].

The role of restrictive gender norms as a critical barrier to achieving gender equity has been gaining interest in many sectors in Nigeria [11]. For instance, recent research has examined the association of gender norms with health outcomes such as intimate partner violence, modern contraceptive use, and institutional deliveries [[12], [13], [14]]. More proximal to maternal, infant, and young child nutrition (MIYCN), recent research demonstrated that harmful gender norms, similar to those that limit women’s mobility and influence providers’ gender-discriminatory attitudes, serve as a barrier to women’s access to healthcare services [15,16] and that food distribution patterns also favor men’s preferences in households that observe traditional gender roles [17].

Understanding the linkages between gender norms and nutritional outcomes is complicated by the multiple determinants of nutrition. In 2023, UNICEF called out the role of harmful social and gender norms as 1 of 8 principal and interacting drivers blocking progress on nutrition [1]. Nutritional status is affected most proximally by dietary practices and availability and access to nutritious foods, nutrition services and healthcare services, but also by domestic division of labor, family and community support, and access to economic opportunities, land, clean water, hygiene, and sanitation; all of which are affected by gender norms and practices [1,18]. Programming in the nutrition sector has begun emphasizing the role of norms in nutrition-related behaviors, including purchasing foods, women’s dietary practices, breastfeeding, feeding young children, and intra-household food distribution patterns [17,[19], [20], [21], [22], [23], [24], [25], [26]]. Yet, to design and implement effective norms-shifting actions to facilitate improvements in nutrition, greater understanding is needed of which gender norms influence which nutrition behaviors, how they influence those behaviors as well as where the norms show signs of being amenable to change [24].

To address this gap in Nigeria, this paper seeks to identify and analyze the influence of gender norms within the family and community that impact MIYCN. The results will distill actionable insights for programming and policy, in Nigeria and globally, to address gender norms to alleviate persistently high rates of malnutrition among women and children.

## Methods

This paper analyzed data from a larger cross-sectional descriptive study designed to obtain an in-depth understanding of the factors leading to MIYCN and related health and water, sanitation, and hygiene (WASH) practices in Nigeria during the first 1000 d from conception until a child’s second birthday [27]. Commissioned by UNICEF Nigeria, the larger study was conducted over October–November 2022 in 1 urban and 1 rural community in each of 6 states (Niger, Kano, Gombe, Oyo, Enugu, and Cross River), 1 per Nigeria’s 6 geopolitical zones. Locations were identified purposively after considering health, nutrition, WASH, and gender indicators, in addition to demographic representation, security concerns, ongoing research, and priorities of UNICEF and the Nigerian Ministry of Health. The study’s primary participants were pregnant women and mothers of children aged <2 y, but also included “behavioral influencers.” The behavioral influencers included in this study encompassed community members providing MIYCN services, information, and support: facility-based and community health workers, fathers, grandmothers, and mothers-in-law. Because this study was a uniquely qualitative inquiry, a purposive nonprobability sampling approach was used to select study participants, engaging community stakeholders and gatekeepers to help identify potential participants. The Nigerian National Health Research Ethics Committee and FHI 360’s Protection of Human Subjects Committee approved the protocol. Data collection was conducted by experienced data collectors who were hired and organized by the Nigerian firm, InSiGHt Health Consulting. The data collection guides were translated into Yoruba, Hausa, and Igbo and the data collectors were trained on the study protocol, research ethics, and other study specifics.

This paper draws from a subset of qualitative data collected as part of 54 focus group discussions (FGDs) and 48 go-along interviews. The subset included interviews and FGDs where respondents were probed about the roles and responsibilities of mothers and fathers/husbands and wives in the MIYCN behaviors of interest. The “go-along” method or “walk-along” interview method was selected for this study because it is a form of in-depth qualitative interview method during which researchers are able to observe behaviors and the context in which they occur while simultaneously discussing the observed behaviors and context with participants [28,29]. Go-along participants were a subset of FGD participants who, during the FGDs, seemed engaged and likely to be knowledgeable about the go-along interview focus behaviors. Go-along interviews were conducted in homes, markets, and healthcare facilities where participants were observed and asked about their behaviors respective to each location (i.e., food preparation and child feeding in homes, food purchasing in markets, and use of healthcare services in healthcare facilities). Data collectors used checklists and notes to record participants’ actions and responses to questions.

The FGDs used a combination of questions and a participatory activity. Participants were asked what behaviors were considered socially typical and/or approved of in their own communities around maternal nutrition; infant and young child feeding; use of child and maternal health services (i.e., antenatal care, delivery, postnatal care, sick child); and paternal involvement in childcare and infant and young child feeding. In the participatory activity called Gender Boxes, participants generated lists of tasks and decisions that are undertaken by mothers, fathers, or both. Facilitators then engaged participants in discussing the differences, similarities, and any overlap in these lists.

The analysis for this manuscript was conducted using the transcriptions translated into English of the audio recordings of go-along interview and FGD discussions. A codebook was created of structural codes from both interview guides and the study domains of interest. Codes were also included for geographic region to explore potential regional variations in themes. Four analysts first coded transcripts using Dedoose, then summarized the data for each research question using an Excel analysis matrix with all codes in the codebooks [30]. Each transcript was coded by 1 coder initially and then coded transcript sections were placed in an Excel spreadsheet and further analyzed and coded as needed by the Principal Investigator and manuscript leads. There were few coding disagreements and discrepancies were resolved on team calls.

As participant discussions were semistructured and open-ended, not every topic emerged in every discussion and resulting transcript. Therefore, exact numbers of participants or transcripts are not presented; general quantifying language such as “many” or “some” is used. We present verbatim text as much as feasible in this paper to best present participants’ own words. To ensure the credibility and authenticity of the findings, all selected themes and quotes were discussed on team calls with Nigerian members of the data collection and study implementation team.

This analysis was informed by social norms theory and guided by CARE’s Social Norms Analysis Plot framework [31]. In social norms theory, the term “gender norms” (or “gendered social norms”) refers to the subset of social norms that define acceptable and appropriate behavior for men, women, girls, and boys [32]. We sought evidence of gender norms, as well as of related social sanctions and norms exceptions. Consistent with the Social Norms Analysis Plot framework, these 2 additional aspects aid the understanding and design of norms-shifting. “Social sanctions” refers to the “rewards (positive sanctions) or punishments (negative sanctions) enacted by a social group on individuals engaging in a behavior,” and are important to understand given that individuals and groups are motivated to follow or comply with social norms out of concern for receiving positive or avoiding negative social sanctions [33]. “Exceptions” describe the circumstances when it is considered acceptable to deviate from the norm and can serve as important entry points for norms change. We generated a list of all of the social sanctions and exceptions mentioned by study participants and then further assessed which were discussed as applying to both men and women compared with just to men or just to women.

## Results

This analysis includes 503 female (67%) and male (33%) participants in the FGDs or go-along interviews. The majority were aged 20–39 y (59%) and there was nearly equal representation of the Northern (49%) and Southern (51%) states as well as Christians (44%) and Muslims (55%).

Our analysis identified 3 predominant gendered social norms that influence women’s and men’s engagement in recommended MIYCN behaviors, as well as exceptions to the norms and the sanctions for violating these norms.

### Norm 1: gendered delineation of household tasks

Across communities, the FGD listing and discussion of male and female household responsibilities revealed strong gender-delineated household roles, with no discernible distinctions across geographies and religions. Fathers were described as responsible for providing food, money, and clothes for the family, and paying school fees and medical bills. One FGD ascribed the following tasks to men:

*“The father provides food for the family, provides shelter, pays the children’s school fees, teaches his children good character, pays the children’s hospital bills and impregnates the mother.”—FGD male participants, North Central*

Participants largely agreed that men’s tasks exclude caring, feeding, and cleaning children. Mothers are always responsible for the children and all household cooking and cleaning. Many male and female participants made statements like the following:

*“It is the responsibility of a woman to fetch water and wash plates and to prepare food and to do everything. These are the responsibilities of a woman.”—FGD participant, male, South West*

“*No, it is not the work of the fathers to do that [care for the child]. A man cannot cook for the family or bathe his children. The woman should do that. But if a man loves the wife, he can assist in the house chores though it doesn't mean that they are his task*.*”—FGD participant, female, South East*

A few household tasks are considered gender-neutral or are shared by spouses/partners. The majority or participants said both parents are responsible for children’s discipline, moral upbringing, and religious practices (e.g., praying, attending the church or mosque). Some participants felt that fathers, as well as mothers, are responsible for preparing and taking children to school and helping them study.

“*Like in teaching their kids good morals, we talked about how the kids are well brought up and both parties contribute - that is both the father and mother*.*”—FGD participant, female, North East*

In the go-along interviews, participants gave mixed responses when asked who typically purchases the household food: some stated that one spouse/partner predominantly goes to the market, whereas others said it depends on who is available or what is being purchased.

“*My husband has the duty of purchasing food items like rice, beans, yam, potatoes. He purchases those ones, but when it comes to purchasing what will be used for preparing the food, then I will be the one to go and purchase them. Like when I want to prepare abacha, I will be the one to go to the market to buy what I need for it*.*”—Go-along participant, female, South East*

#### Exceptions

Some participants described circumstances where men might defy gender norms and perform tasks commonly ascribed to women. The most common scenario was a husband helping his sick or pregnant wife with household duties. A minority of participants noted that if the wife and husband are at peace in the home, the husband will feel comfortable helping her. One participant said that if the wife works outside the home, her husband may help with domestic tasks. The possibility that men might go against gender norms by helping wives with tasks was discussed more frequently in the southern regions.

“*It is a responsibility of a woman to fetch water in the house, to wash plates and to wash all, to do all things. But sometimes maybe the woman is pregnant or is her time then we need to help her. Maybe it has reached eight months and her time to deliver has come. She will not be able to bend, to do most things at that time. The man must take care of the woman at that time*.*”—FGD participant, male, South West*

Some participants also shared situations when women may take on typically male roles. In one example, it would be socially acceptable for a wife to take on more male-associated tasks if her husband is absent or has died.

“*Most women that do the work of their husbands in the home are those that their husbands have died. So, people will not blame them as such*.*”—FGD participant, female, South East*

Several FGDs noted that when women earn more money than men, they often become responsible for additional household tasks and decisions, contrary to normative expectations. Some participants said the shift in roles should be kept private as others in the community may judge them.

“*There are some homes where the father does not have the requisite skills. If the woman assists in taking the decisions [in those homes], it is not bad as long as she does not neglect the husband in the process. But people around will see the woman as one that controls the husband*.*”—FGD participant, female, South East*

### Norm 2—gendered household decision-making

The second distinct gendered social norm identified through FGDs relates to household decision-making. All participants agreed that men are seen as the primary household decision makers.

“*Starting from the children until when they get old, it is the man that makes the decisions in the house. So it is not all families that women are second in command. The man is the first and second in command*.*”—FGD participant, female health worker, South South*

Men are responsible for decisions about their children’s schooling, friends, movement, jobs, and marriage, and their wives’ employment and mobility. Men also are seen to control decisions about their spousal relationship, such as divorce or marrying more wives. Potentially most significant for MIYCN, fathers were described as the decision makers for healthcare. This includes choices on when and where family members can seek care at a health facility for illness, antenatal and postnatal care, and delivery.

“*The husband is the permission giver, [we can only come to this healthcare facility] if he says we should come and if he says otherwise, we cannot. He can tell us the specific health facility he wants us to go to*.*”—FGD participant, female, South West*

The most common reason mentioned for why men typically control household decisions about food purchasing and healthcare utilization was because men control household finances.

“*It is the father that must provide the money for his wife to buy food. If the father drop money for his wife, he has the right to give orders to his wife that this is what I want to eat in the house today… If there is no money with the father, there is no order that the father can give. Yoruba have a proverb – they say ‘an elder that does not have money will become a lout.’ So if there is no money with the father, it is compulsory he will become a lout in the house. But if there is money, as his responsibility, it is the father that must decide what the wife will cook in the house*.”*—FGD participant, female, South West*

Most FGDs expressed that mothers typically make few to no household decisions. A sizable number of participants, especially in the Northern regions, attributed this limited female role to men being the sole decision makers as the leads of the home.

“*The mother does not have any decision on her home but what the husband says. Due to cultural beliefs women don’t take any decisions on their own*.*”—FGD participant, male, North Central*

“*Whatever will happen in his house has to be with his agreement. If he says they should process millet, it should not be corn. You cannot go out without his utter permission. If you want to live in peace with him, it has to be that you must obey his instruction*.*”—FGD participant, male, North West*

#### Exceptions

There were a few select types of decisions that participants (FGDs and go-along) considered to be joint or could be made by either spouse/partner. These exceptions to male-dominated decisions centered on child rearing, food purchasing, meal preparation, and household food distribution.

“*It is the woman that the children move closer to, and it is there that they really will be collecting lessons …. Truly, the man is the head of the house and can say let's do it and they will agree. But about the children, the woman has a lot of authority*.”*—FGD participant, male, South West*

“*On who decides on food purchase or what to cook, mothers too have say on this, because they know what is in the store and what is not there. Even if the husband says today, we should eat this or that, the mother can change this decision when she looks at what she has in her store or kitchen. So, mothers have power on the type of food to be cooked in the house*.”*—FGD participant, female, North West*

The degree of female involvement in food purchasing decisions varied between the FGDs and go-along interviews. Women were described as sole decision makers more often in the FGD Gender Box activity, whereas the go-along interviews indicated that food purchase decisions were joint between spouses/partners. Joint decisions took 2 forms; husbands indicating how much to spend and wives selecting which food items to purchase, or spouses/partners choosing together.

“*What made them my duty is that when my husband brings money, it is my responsibility to know what I will purchase. It is his duty to provide money then I will go and purchase whatever I want to prepare*.”*—Go-along participant, female, South East*

Participants expressed mixed responses on the decision of who eats first in the household. Many widely agreed men eat first when they are at home; however, a notable set of participants described this as a joint decision, with the husband/father having some say as he pays for food, but the wife/mother also having a say because she prepares food.

“*The decision of who eats first in the family is in the hands of the wife because she is the one that cooks*.”*—FGD participant, female, South South*

### Norm 3—women’s restricted mobility

The third gendered social norm that arose was women’s restricted mobility, and how it may hinder women’s ability to engage in recommended nutrition and health practices. In discussions, many participants made statements such as,

“*The father decides the specific time to close his house gate. The father decides and approves where his wife can go*.”*—FGD participant, female, North Central*

“*Because of cultural beliefs, the woman … she needs permission before she goes. It can lead to problems when she goes out without his permission, so she needs to seek permission before*.”*—FGD participant, male, North East*

This norm was discussed most frequently in relation to women’s ability to seek healthcare and access health facilities, either for themselves or their children.

“*The neighbors tell me to take my child to the hospital when he is sick. But his father has refused so it is not my fault*.”*—FGD participant, female, South West*

#### Exceptions

Notably, women’s need to obtain male permission to go outside the home was largely constrained to healthcare seeking. Across go-along interviews and FGDs, interviewers observed, and participants frequently discussed, that women’s travel to other community locations, such as markets to purchase food, did not require permission. Male and female participants also agreed that under exceptional, emergency circumstances (e.g., serious illness, going into labor) it is acceptable for women to seek and travel for healthcare without permission from their husbands or male household members.

“*Before the woman or the wife does something, she must get the authority from oga (influential senior or boss), except it is urgent in some cases. When my child is sick, my wife does not need to get my permission*.”*—FGD participant, male, South South*

“*Labor is an emergency situation that does not need permission from anybody before going… If the husband is unsupportive, the woman might seek his permission but go straight to the hospital to save her life and that of her baby*.”*—FGD participant, female, South East*

### Sanctions

Throughout the discussions, participants described a variety of social sanctions that are imposed by household or community members upon those who do not comply with gendered norm. Sanctions can vary in severity from loss of respect to social stigma to more severe repercussions such as divorce, violence, or police action. Most participants felt that the main sanctions motivating fathers and mothers to conform to gender norms are those meted out by the community: a desire for community respect, for themselves and their families (i.e., positive social sanctions), and to avoid shame, disrespect, and shunning from community members (i.e., negative social sanctions).

“*When they come to realize that what they are doing is totally not accepted in [community name] and most persons stop respecting them, they will want to ‘stay inside the box’ (adhere to gender norms).*”*—FGD participant, male, North Central*

**FIGURE 1:**
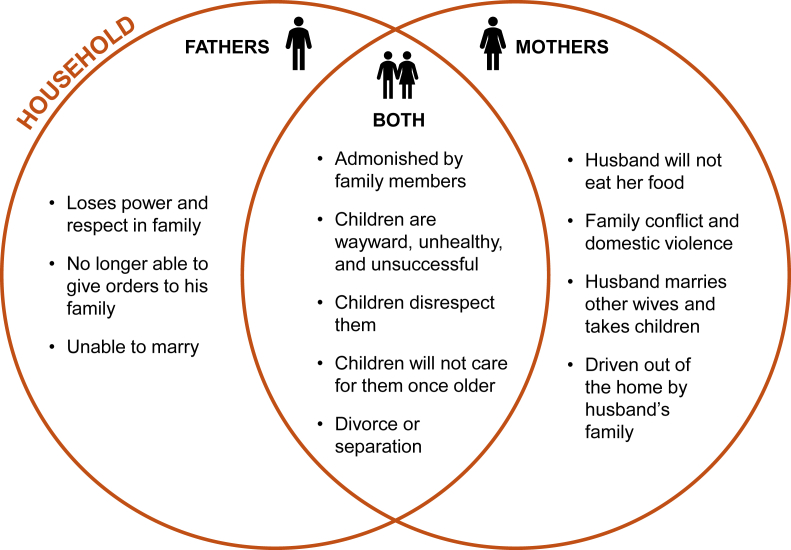
Household level sanctions for fathers, mothers, and both for violating gender norms.

**FIGURE 2:**
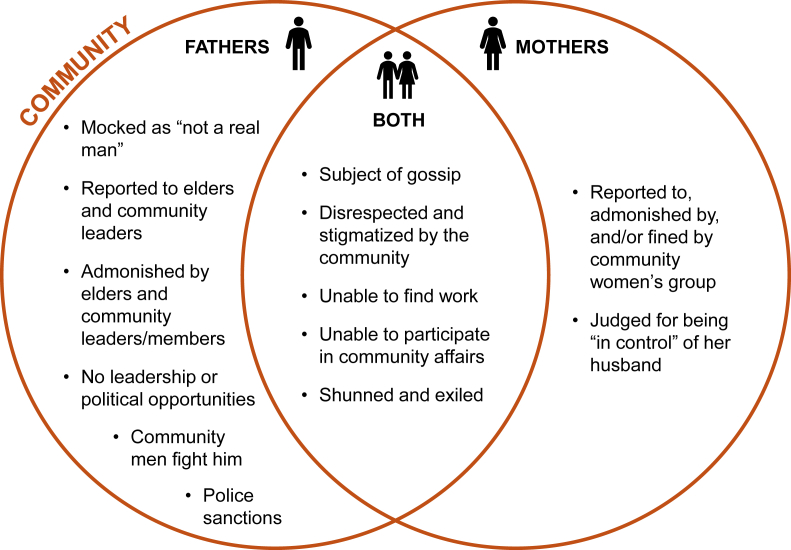
Community level sanctions for fathers, mothers, and both for violating gender norms.

Both men and women can face social sanctions for going against gender norms; however, the source of sanctions largely differs for men and women. Sanctions for women are most frequently meted out within the household, such as with domestic violence, being sent away from the home, or the husband marrying a new wife (Figure 1). Men are more likely to face sanctions from community members and leaders (Figure 2).

At the household level, the most frequently discussed sanction was from one’s children. Both male and female participants feared that if they transgressed a normative behavior their children would no longer care for them when in old age.

“*She will be alone and the children will also neglect her because she did not do well earlier. She will not enjoy the fruit of her labor*.”*—FGD participant, female, South West*

“*It is the father that takes care of the children that will enjoy the fruit of his labor. But anybody who refuses to take care of his children is irresponsible, especially the alcoholics, and the children will desert him*.”*—FGD participant, female, South West*

Divorce and separation were also discussed as potential sanctions for men and women at the household level; however, single parenthood due to divorce or separation was only reported as something women may face.

“*At the end of the day, mothers like that become single mothers, the father might abandon the mother and child, divorce the mother or drive her out of his house, and later she becomes a single mother*.”*—FGD participant, female, South West*

At the community level, both women and men who transgress social norms could face admonishment, disrespect, loss of work, stigma, and exile from the larger community. For men, however, the reported repercussions are more numerous and serious at the community level, coming from community members and institutions, such as community leaders, police, and elders.

“*If you want to do politics, your community they will first look at where did he come from, how does he do to his wives at home? This one cannot lead us because he cannot control his house. Is this who will we appoint as leader? This is what causes setbacks to anything you want to do in the community--either in the community, in the environment, in politics, Not being a good father to your wife and children, it causes problems later for you*.”*—FGD participant, male, South West*

“*If he behaves the way he’s not supposed to behave in our community, they will take him to the palace, our chief will question him., If you don’t behave as a man and father, they will drive you out of the community*.”*—FGD participant, female, South South*

Many participants anticipated that if a man disregarded gender norms, he would also lose power to make decisions in his household, which would result in widespread community stigma.

“*An irresponsible father won’t be respected and because he is irresponsible, he will not be able to give commands to the child or the wife, or decide for the family. He will be seen as an unimportant being because he does not do his roles*.”*—FGD participant, female, South West*

Numerous participants also conveyed the concern that men will be mocked and insulted for appearing under his wife’s control and thus, “not a real man.”

“*They will say that the man is a woman wrapper. They will say that the man is a stupid man, that there is no real man that will stoop so low as to wash his wife's clothes*.”*—FGD participant, female, South East*

## Discussion

In our search for gender norms affecting specific, individual MIYCN behaviors, we found 3 cross-cutting gendered social norms that simultaneously impact multiple nutrition outcomes across the 1000-d period. These meta-norms center on gendered household responsibilities, decision-making, and mobility. Participants described strong traditional gender ideologies and sanctions that uphold these restrictive norms and, therefore, in turn can inhibit a variety of recommended MIYCN practices in the areas of food cultivation, purchasing and preparation, antenatal care, institutional delivery, postnatal care, breastfeeding, complementary feeding, child growth monitoring, and care seeking for child illness. Our findings provide important insights into the strength of these meta-norms in terms of how they are regulated as well as where there may be exceptions to the norms that could indicate which norms are starting to shift and could serve as entry points for future gender transformative and/or norms-shifting approaches for MIYCN.

The aim of this analysis was to tease out behaviorally specific gender norms affecting recommended practices for optimal MIYCN, such as whether it is normative to purchase and consume nutritious foods or to attend antenatal care; instead, we found meta-norms. Meta-norms are particularly influential norms that, “connect with deeply rooted determinants [and] operate at a more profound level of society and influence multiple behaviors” [34]. Our findings are valuable for future nutritional interventions, as a focus on meta-norms may prove simultaneously more impactful and broad reaching.

Our analysis explored possible geographic distinctions in norms as previous reports have indicated differences in gender and MIYCN issues across regions in Nigeria [9,35]. We found participants in the North predominantly reported women’s lack of decision-making power and almost exclusively participants in the South describing how men might go against gender norms by helping their wives with household tasks. This distinction may be an indication of norms being less restrictive and/or beginning to change in the southern part of Nigeria. However, generally we did not detect differences in themes geographically and the 3 meta-norms simultaneously emerged in FGDs across Nigeria’s 6 geopolitical zones. The combination that these norms were meta-norms and were widespread throughout Nigeria suggests that collaboration between nutrition and other health and development sectors on policies and programs that seek to address gender equity broadly in Nigeria will be an expedient route to impacts on an array of MIYCN outcomes.

Study participants noted several situations in which exceptions are made for community members to act in ways that are not considered typical or approved of. For instance, women may travel to healthcare facilities alone and/or without permission in emergencies or step into traditionally male roles when their husbands are absent or deceased. In some cases, it is also acceptable for women to be involved in decision-making surrounding child rearing, food purchasing, meal preparation, and household food distribution. Although many of these exceptional circumstances seem intuitive, documenting and exploring these context-specific exceptions to gendered norms is useful as they may be harbingers of shifting norms and can be employed as points of discussion with community members to point out that there is precedent for women breaking with norms and experiencing success.

Noteworthy in our findings were the discussions of how women earning money shifted gendered decision-making in households. Previous research has examined the impact of women’s ownership of assets such as land and agricultural products on household dynamics, and found empowering women in agrifood systems results in greater food security and improved nutrition for the entire household [36,37]. Previous research has also shown that although men tend to focus on cash crops, women tend to have less-lucrative positions in agrifood systems and tend to be responsible for cultivation for the household’s nutritional needs as an extension of their domestic labor responsibilities [38,39]. Our findings, combined with this previous research, reinforce the need for multi-component and cross-sectoral gender-transformative nutrition interventions. Given women’s central role in food preparation and cultivation for the household, nutrition interventions need to include components that bolster women’s access to agricultural and nutritional knowledge, technical skills, and resources so as to augment food security and nutrition at the household level. Simultaneously these interventions also need to bolster women’s economic opportunities as a lever to greater gender transformative change within households, agrifood systems, and the larger economy, allowing for more equitable distribution of the domestic workload and greater participation of women in more lucrative positions in agrifood system value chains as well as other workforce roles. Notably, results of a recent landscaping of the social norms literature and programs in Nigeria found only 1 article examining the effects on nutrition outcomes of shifting gender norms through women’s economic empowerment [40]

Our findings also provide useful insights regarding how meta-norms are regulated within communities and families in Nigeria. Many of the sanctions individuals could face for acting outside of expected norms are quite severe, such as domestic violence, police action, and being driven out of the home. This analysis also revealed that the source of sanctioning is largely different for men and women; women’s sanctions are most often meted out by their household, whereas men’s are typically imposed by the broader community. Future interventions and programming must be mindful of these repercussions, to both ensure the safety of their participants and to better engage family members and broader communities in the transformation of gender norms. To specifically target norms affecting men, the involvement of community organizations, leaders, and elders is critical to shift how they react to and sanction male community members.

Consistent with previous research, our findings showed that traditional normative roles are restrictive for men as well as women and, in particular, gendered delineation of household tasks limits fathers’ involvement in feeding children, thereby, distancing them from thinking about the nutritional needs of family members [38,41,42]. Encouragingly, for both men and women, discussions with communities indicated that they are socially cohesive and that community members are involved in sanctioning and, thus, regulating each other’s behaviors. Given identified sociocultural patterns of noting and rewarding other community members’ behavior, 1 idea for future interventions may be to encourage recognition and celebration at the community level of mothers, fathers, and families practicing optimal behaviors, such as cultivating and feeding their families nutrient-rich diets and seeking healthcare during pregnancy and delivery. Openly celebrating such behaviors may serve as a strong motivator for other families to follow and has been employed by programming elsewhere [21].

It is important to note several distinctions in the design, implementation, and setting of this research. Our study employed uniquely qualitative approaches to data collection, with the aim of providing contextual insights and perspectives rather than describing quantitative patterns, rates, and trends. Although efforts were made to select a diverse sample of communities for this assessment, in a nation as large and varied as Nigeria, it is not possible to generalize themes found in small qualitative sample sizes to all of the geographic and cultural contexts represented within the country.

Nigeria is contending with levels of undernourishment and food vulnerability that are on the rise and have recently been the subject of urgent calls for action [1,43]. In Nigeria, impending demographic and climate challenges and persistent gender inequalities make these calls particularly pronounced. Globally, the widening food security gap between men and women, and the growing “triple threat” of undernutrition, overnutrition, and micronutrient deficiencies among women, calls for interventions that both address multiple nutritional challenges simultaneously and more explicitly tackle the links between gender inequality and nutrition [1,44]. The findings from this analysis contribute to the capacity of the Nigerian government and implementing organizations to address Nigeria’s nutritional challenges by deepening understanding of and providing actionable insights regarding the connections between gender equity and nutrition. Policies and interventions that seek to shift gender meta-norms are likely to achieve impacts and improvements across multiple behaviors and health and development outcomes simultaneously.

## Author contributions

The authors’ responsibilities were as follows – EC, CM, MG: wrote the manuscript; IO, NH: developed the study concept; EC: designed the study with input from KL, IO, and NH; EC, CM, KL: analyzed the data; NM-O: lead the data collection for the study; and all authors: read and approved the final manuscript.

## Data availability

Data described in the manuscript, code book, and analytic code will be made available upon request.

## Funding

This work was funded by UNICEF Nigeria, who was involved in the study design, implementation, and interpretation of data.

## Conflict of interest

The authors report no conflicts of interest.
